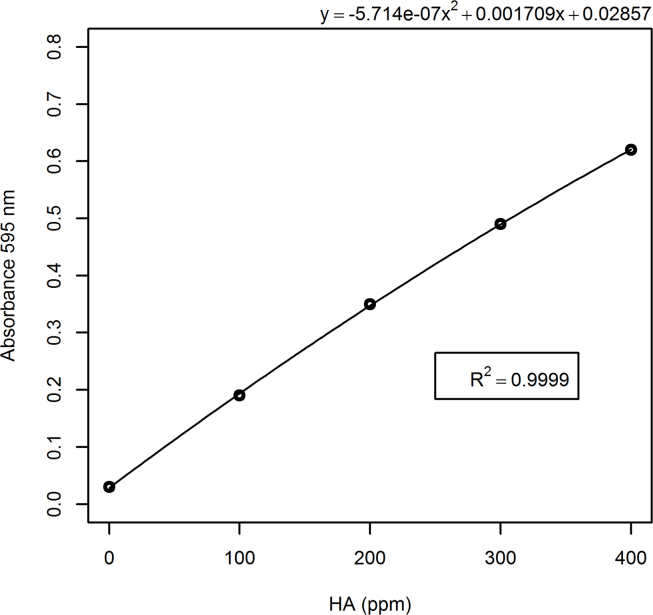# A comparison of two colorimetric assays, based upon Lowry and Bradford techniques, to estimate total protein in soil extracts

**DOI:** 10.1016/j.soilbio.2013.08.017

**Published:** 2013-12

**Authors:** M.A. Redmile-Gordon, E. Armenise, R.P. White, P.R. Hirsch, K.W.T. Goulding

**Affiliations:** Rothamsted Research, Harpenden, Herts AL5 2JQ, UK

**Keywords:** Total protein determination, Colorimetric protein assay, Polyphenol interference, Glomalin related soil protein, Humic acid, GRSP, BRSP, EPS, Soil microbial biofilm, Extracellular polymeric substances

## Abstract

Soil extracts usually contain large quantities of dissolved humified organic material, typically reflected by high polyphenolic content. Since polyphenols seriously confound quantification of extracted protein, minimising this interference is important to ensure measurements are representative. Although the Bradford colorimetric assay is used routinely in soil science for rapid quantification protein in soil-extracts, it has several limitations. We therefore investigated an alternative colorimetric technique based on the Lowry assay (frequently used to measure protein and humic substances as distinct pools in microbial biofilms). The accuracies of both the Bradford assay and a modified Lowry microplate method were compared in factorial combination. Protein was quantified in soil-extracts (extracted with citrate), including standard additions of model protein (BSA) and polyphenol (Sigma H1675-2). Using the Lowry microplate assay described, no interfering effects of citrate were detected even with concentrations up to 5 times greater than are typically used to extract soil protein. Moreover, the Bradford assay was found to be highly susceptible to two simultaneous and confounding artefacts: 1) the colour development due to added protein was greatly inhibited by polyphenol concentration, and 2) substantial colour development was caused directly by the polyphenol addition. In contrast, the Lowry method enabled distinction between colour development from protein and non-protein origin, providing a more accurate quantitative analysis. These results suggest that the modified-Lowry method is a more suitable measure of extract protein (defined by standard equivalents) because it is less confounded by the high polyphenolic content which is so typical of soil extracts.

## Highlights

• Bradford and Lowry techniques for measuring protein in soil extracts were compared.
• We found polyphenol content affected Bradford estimations substantially more than Lowry.
• Citrate (often used to extract soil protein) was not found to be problematic for the Lowry assay.
• Using the Lowry microplate method, analytical response due to polyphenol and protein is ascribed.

## 1 Introduction

All methods of total protein estimation are subject to artefacts when analysing extracts of soil and so are best thought of as being ‘semi-quantitative’. The selection of analytical method depends very much on how we choose to define soil protein, and the analytical resources at our disposal (Gillespie et al., 2011). For example, Roberts and Jones (2008) favoured hydrolysis of protein followed by chromatographic quantification of the constituent amino acids. While the analytical stages of hydrolysis approaches are highly tolerant of interference from humic substances, there are several problems. Firstly, partially humified organic molecules are likely to release some amino acids by hydrolysis, secondly, some proteinogenic amino acid residues (e.g. tryptophan and cysteine) are destroyed in the hydrolysis step, and furthermore some peptide bonds are not successfully hydrolysed, particularly bonds of hydrophobic residues such as valine, isoleucine and leucine (Roberts and Jones, 2008).

In contrast, colorimetric methods suffer interference from humic substances directly, seriously limiting their accuracy (Nannipieri and Eldor, 2009). However, colorimetric methods are still frequently used for relative comparison between treatments as they are rapid and affordable, do not require a hydrolysis step, and frequently show good correlation to more expensive and time-consuming techniques (e.g. Gillespie et al., 2011). Soil extracts usually contain large quantities of humified organic matter, which is characterised by high polyphenolic content (Martens et al., 2004). This has been demonstrated to erroneously increase estimates of extracted protein using the Bradford assay (Whiffen et al., 2007). Furthermore, this interference cannot be assumed to be constant because the size of the polyphenolic pool can be variable under different managements (Martens et al., 2004), increased for example through secondary metabolism of fungi (Haider et al., 1975), or decreased through termite metabolism (Ji et al., 2000) and microbial priming effects (Kuzyakov et al., 2000).

Low molecular weight phenolic fractions have previously been removed from aqueous solutions using polar solid-phases (Ferri et al., 2011) however, this approach is also likely to cause the removal of dissolved protein (Bianchi et al., 1996). Giagnoni et al. (2011) also reported protein losses occur using gel filtration to purify extracts containing humic substances. Removal of humic complexes would also be problematic because polymerisation of extracellular proteins by humic molecules is an inevitable and natural process in most soils (Burns et al., 2013). The quantities of smaller humic-peptides are also highly variable between extracts of different soils (Bonmati et al., 2009). Furthermore, irreversible macromolecular associations are suspected to form in response to extraction (Schmidt et al., 2011). Reducing the magnitude of artefacts arising from polyphenolic content is therefore a reasonable alternative to attempted removal of the polyphenolic fraction.

The Bradford assay (Bradford, 1976) has become the colorimetric method of choice, owing principally to its high sensitivity, perceived linearity, and the speed of analysis (Sapan et al., 1999). The Bradford assay relies on interactions between basic amino acids residues (primarily arginine, lysine and histidine) with the Coomassie brilliant blue G-250 dye (CBB) in an acidic matrix. The binding of CBB to proteins (or interferands) results in a spectral shift to the blue form of the dye. In contrast, the Lowry assay (Lowry et al., 1951) functions in alkaline conditions, and involves two steps: 1) the Biuret reaction: based on the reduction of Cu^2+^ which then binds to protein forming a Cu^1+^ peptide complex, and 2) subsequent reduction of the Folin–Ciocalteu reagent by this complex (Smith et al., 1985). In the original format proposed by Lowry et al., (1951) the Lowry assay also gave a false indication of protein in the presence of polyphenols, which both reduce the Folin–Ciocalteu reagent, contributing to absorbance in the same region of the spectrum for protein complexes (∼750 nm).

Colorimetric investigations of soil extracts are also strongly affected by physical interferences (scattering), and physico-chemical effects from suspended clays (sorption). Centrifugation at 3000 × *g* does not completely remove the extra-fine clay fraction, which is the most active in these sorption process (Lozzi et al., 2008). In the aforementioned study, the Lowry assay was the only method to give correct protein estimates. Besides being highly sensitive to residual clay content, the Bradford assay is highly time-sensitive, with precipitation of protein-bound-dye occurring about 10 min after contact. This introduces limitations regarding the number of samples measurable per run, reducing throughput and speed.

The citrate extraction technique described by Wright and Upadhyaya (1996) is widely practiced, with protein content commonly referred to as glomalin related soil protein (GRSP). The protein content in these extracts was originally proposed to arise from glomalin producing arbuscular mycorrhizal fungi (AMF) of the Glomeromycots, but extracts have since been confirmed to contain large amounts of soil protein from non-mycorrhizal origin (Gillespie et al., 2011, Rosier et al., 2006). Regardless of origin, the use of Bradford's assay to measure either GRSP, or protein in general, generates a false measurement when assayed in the presence of polyphenols which occur both in large and highly variable quantities in soil extracts (Halvorson and Gonzalez, 2006, Roberts and Jones, 2008, Whiffen et al., 2007). Furthermore, the interference from polyphenols is not quantified in the assay procedure. This means that no distinction can be made between the protein and polyphenolic content, and thus references to GRSP or even protein quantified using the Bradford assay can be very misleading (Nannipieri and Eldor, 2009).

Lucarini and Kilikian (1999) found that 2 M citrate caused suppression of colour development, leading to an underestimation of about 19% in the Lowry assay and 5% in the Bradford method. The sensitivity of the Lowry assay to citrate might first appear prohibitive. However, extractions of GRSP use citrate concentrations of only 20 mM (pH 7) or 50 mM (pH 8). Furthermore, a modification to the Lowry assay employed by Frolund et al. (1995) claimed to enable separation of absorbance due to protein and that from the humic fraction, by inclusion and exclusion of copper sulphate from the Lowry reagent. Although this has been successful for biofilm extracts of waste-water sludges, it has not previously been tested with soil extracts.

We therefore compared the routine Bradford assay with a microplate adaptation of Lowry et al. (1951) including the modification described by Frolund et al. (1995) which claimed to enable separation of absorbance due to protein and that from the humic fraction. Increases in total protein content were measured by addition of known quantities of BSA to citrate extracts of 3 contrasting soils. Our aims were to:

i) Determine if citrate is problematic in Lowry microplate assays of soil extracts.
ii) Compare the accuracy of Bradford and Lowry estimations of protein additions to soil extracts (Bovine serum albumin; BSA), both with, and without increasing polyphenol additions (humic acid; Sigma H1675-2).

## 2 Materials and methods

### 2.1 Site description and soil sampling

Three soils of contrasting management were obtained from two experimental sites in Southern England with contrasting chemical and physical properties (Table 1). Soil 1 (Field: ‘Long Hoos’) was under wheat cultivation at Rothamsted Research, Hertfordshire, UK (50°50′ N, 0°25′ W). Soil 1 is classified as a flinty clay loam over clay with sandy inclusions (Batcombe series). Soil 2 and Soil 3 were from Woburn Experimental Farm, Bedford, UK (51°59′ N, 0°35′ W), and classified as sandy Cambric Arenosols (FAO). Soils were sampled both from a bare fallow management area (Soil 2) and from permanent grassland (Soil 3). Composite soil samples were collected in April 2010 using a 2.5 cm diameter auger to a depth of 0–23 cm for the arable and bare fallow soils (Soils 1 and 2) and of 0–10 cm for the grassland (Soil 3). Samples were bulked and stored overnight (10 °C) before sieving moist (<2 mm) and subsequently air-dried in the dark at 25 °C.

**Table 1:** Soil main chemical and physical properties.

Soil no.	Soil type	Management	Organic C (μg g^−1^)	Total N (μg g^−1^)	C/N ratio	pH	Clay %^a^
1	Chromic luvisol	Arable (wheat)	13.66	1.30	10.5	7.18	18–27
2	Cambric Arenosol	Bare fallow	0.30	0.03	10.3	5.53	7.9
3	Cambric Arenosol	Grassland	16.86	1.55	10.9	5.95	8.0

a Data from Avery and Catt (1995).

### 2.2 Soil extractions

Soils were extracted using the ‘easily extractable glomalin’ protocol of Wright and Upadhyaya (1996). Briefly, 8 ml of 20 mM sodium citrate at pH 7.0 was dispensed onto 1 g of air-dried soil in 15 ml polypropylene centrifuge tubes and autoclaved (121 °C) for 30 min. Immediately after autoclaving, the tubes were cooled on ice and centrifuged in a pre-cooled rotor (4 °C) at 3500 *g* for 20 min. The supernatants were decanted and stored overnight at 4 °C for analysis.

### 2.3 Sample and standard preparation

A model polyphenol (humic acid; Sigma H1675-2) and protein (bovine serum albumin; Sigma A7906) were used throughout as standards. Soil extracts 1, 2 and 3 were diluted 7.5, 2 and 12.5 times respectively, with phosphate buffered saline (PBS) to remain within the effective assay range (maximum absorbance < 1.0). BSA standard additions were equal to 0, 20, 40, 60, 80 ppm for the soil extracts, and 0, 25, 50, 75, 100 ppm for standards in PBS alone. These were combined with humic acid (HA) additions of 0, 80, 160, 240, 320 ppm for the soil extracts, and 0, 100, 200, 300, 400 ppm in PBS alone.

### 2.4 Bradford microplate analysis

Protein measurements in extracts and PBS were made using a Bradford assay kit (Bio Rad Protein Assay; Bio Rad Laboratories). To each 50 μL of dilute extract or PBS (three replicates) in microplate (Nunc 442404) were added 25 μL aliquots of PBS containing sufficient BSA and/or HA to give equivalent concentrations (relative to the original 50 μL extract) of 0–100 ppm BSA, and 0–400 ppm HA. Bio-Rad G-250 dye was rapidly mixed with PBS immediately before addition, then immediately and forcefully added with a 12-channel pipette to ensure adequate mixing (Thermo electronic ‘Finnpipette’ set to speed 9). This delivered 50 μL of dye to each well with sufficient PBS to reach a final well volume of 250 μL. Plates were read 7 min later at 595 nm using a ‘Varioscan’ plate reader (Thermo Scientific) set to a read duration of 150 ms per well.

### 2.5 Lowry microplate analysis

A modification of the Lowry assay (1951) described by Frolund et al. (1995) was used to separately quantify the proteinaceous and polyphenolic compounds in each soil extract. The principle of this modification is that the omission of copper sulphate from the reagent enables determination of the auto-absorbance from humic compounds and chromogenic amino acids. Concentrations and volumes of Lowry and Folin–Ciocalteu's phenol reagents (Sigma F9252), were optimised for speed and sensitivity (results not shown) using principles from Miller, 1959, Oosta et al., 1978 and Peterson (1979). Ultimately, a more concentrated reagent compared to that of Lowry et al. (1951) was prepared to maximise assay sensitivity, and reduce incubation time as detailed below.

Lowry reagents were made from three stock solutions at 3.5 times the concentration of the original Lowry macroassay reagent, i.e.; 3.5 g copper sulphate (CuSO_4_·5H_2_O) 100 mL^−1^ H_2_O, 7 g sodium potassium tartrate 100 mL^−1^ H_2_O, and 70 g Na_2_CO_3_ L^−1^ 0.35 N NaOH. The three solutions were combined sequentially in proportions of 1:1:100 (v:v:v), respectively (Reagent A). The second reagent (Reagent B) was made in the same way, except the copper sulphate solution was excluded and volume substituted with deionised water. Three replicates of each sample or standard (50 μL) were added to 2 × 96 well microplates, marked ‘A’ and ‘B’. The volumes of all wells were increased to 100 μL using PBS or standard in PBS, subsequently, 100 μL of reagent A was rapidly injected to wells in plate A using a 12 channel electronic Finnpipette set to speed 9 to ensure good mixing. Reagent B was added to plate B in the same way. Both plates were incubated at room temperature in the dark for 10 min. Folin-Phenol reagent was prepared immediately before the end of the first incubation (2 N diluted 10 fold in H_2_O), and 100 μL subsequently injected to all wells. The plates were then incubated for a further 30 min (room temperature, dark) before reading at 750 nm for 150 ms per well.

Two absorbencies per sample were thus obtained: ‘*Abs*_*A*_’ and ‘*Abs*_*B*_’ for the respective reagents. From these absorbencies, theoretical absorbance due to protein (BSA equivalents) was calculated as ‘*Abs*_*protein*_’. Absorbance due to ‘humic substances’ (specifically ‘humic acid equivalents’ or HAE) is presented as ‘*Abs*_*humic*_’ as per the following formulae given by Frolund et al. (1995):

$$Abs_{protein}=1.25\left(Abs_{A}-Abs_{b}\right)$$

$$Abs_{humic}=Abs_{b}-0.2Abs_{protein}$$

### 2.6 Assessing the effect of citrate upon the Lowry microplate method

Samples of 100 μL with standard additions of BSA and citrate were prepared by combining 25 μL of 2.5× diluted soil extract (soil 1) with 25 μL PBS containing 80, 160, 240 and 320 ppm BSA, in three replicates, in microplates ‘A’ and ‘B’. Citrate (20 mM) was added to these samples (0, 10, 20 or 40 μL) then made up to 100 μL final volumes with PBS buffer. The final soil extract dilution was thus 10 fold, with concentrations of protein being +0, +20, +40, +60, +80 ppm. The citrate concentrations investigated were therefore 1, 2, 4 and 5 times the typical assay concentration. The modified Lowry procedure described in paragraph 2.5 was then followed. Citrate additions to PBS buffer (no soil extract), and PBS with 160 ppm humic acid (no soil extract) were also prepared for contrast.

### 2.7 Accuracy of protein estimations

The effect of humic acid on protein signal was assessed for each extract of soil (Table 1) and PBS alone, in factorial design, including extract type, BSA and HA addition.

### 2.8 Statistical methods

#### 2.8.1 Citrate effect

Changes in *Abs*_*protein*_ due to BSA and citrate additions were examined by multiple regressions using Residual Maximum Likelihood (REML). The effect of increasing additions of citrate upon *Abs*_*protein*_ over all additions of BSA and citrate is presented for the different analytical matrices.

#### 2.8.2 Protein specific absorbance and humic acid effect

The known additions of BSA were compared with the measured increase in protein concentration of the samples. Regression analysis was used to compare the strength of the impact of HA additions on the intercept and slope of the calibration in buffer and soil extracts.

## 3 Results

### 3.1 Citrate effect on Lowry analysis

**Fig. 1:**
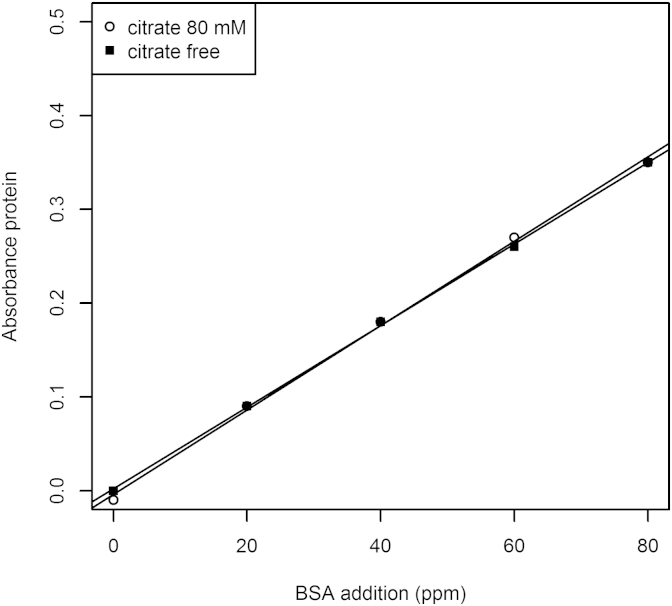
Negligible effect of citrate upon Lowry estimation of standards in PBS containing 160 ppm HA.

**Fig. S1:**
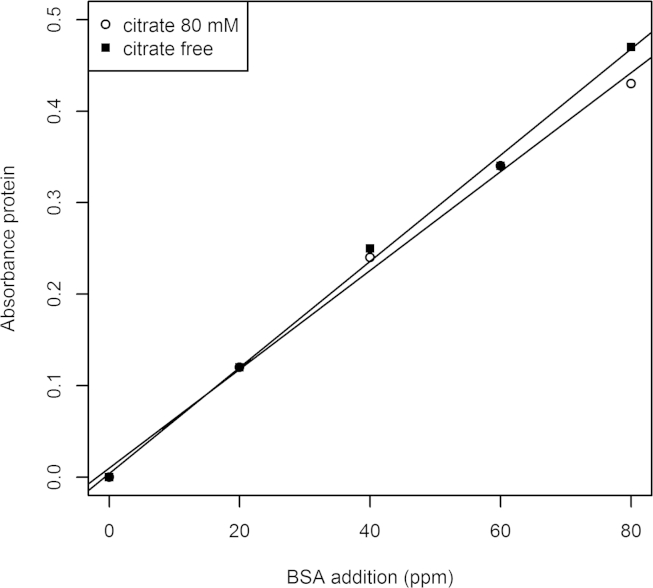
Effect of citrate upon Lowry estimation of a) standards added to PBS, b) standards added to soil extract 1.

Regression analysis of the additions of BSA with increasing additions of citrate to assay buffer, buffer plus 160 ppm HA, and soil extract revealed negligible impact of citrate upon *Abs*_*protein*_. The resulting calibration curves of *Abs*_*protein*_ against BSA additions to soil extract and buffer plus HA were thus almost indistinguishable at the highest and lowest concentrations of citrate tested (Fig. 1). Comparing the linear regressions of *Abs*_*protein*_ as a function of BSA in PBS containing neither soil extract nor HA addition, a very small difference is discernible (Fig. S1a).

Graphical representation of the effect of all citrate additions upon *Abs*_*protein*_ ranging from +20 mM to +80 mM over all BSA additions requires three-dimensional representation (inclusion of *z* axis), and when plotted, the difference is visually indistinguishable. Therefore mean slopes of calibration curves along the *z* axis are given in Table 2, showing negligible effect of citrate on all three extracts.

**Table 2:** Regression coefficients of multiple regressions for citrate additions given with standard error (s.e.).

Analytical matrix	Mean *Z* slope Abs protein as f [citrate]	s.e.
PBS	−0.000266	0.000146
Soil extract in PBS	+0.000294	0.000146
HA spiked PBS (160 ppm)	+0.000052	0.000146

A mean *f* [citrate] (*Z* slope) of −0.000266 for PBS shows that increasing additions of citrate to PBS marginally represses colour development from protein added, as seen by Ji (1973). The extent is negligible however in the range of citrate concentrations likely to be used for soil extraction (Fig. S1b). The negligible effect of citrate is put in perspective by comparison with the matrix effect of HA in PBS (Table 3). It is evident here that the addition of 160 ppm HA to the buffer clearly suppressed *Abs*_*protein*_, changing absorbance from 0.005573 per unit BSA, to 0.004399 per unit BSA (about 20%). Similarly, in soil extract, *Abs*_*protein*_ is 0.004485 of BSA addition (again, about 20% less compared to the response of BSA additions to PBS). The suppression of colour development due to protein (*Abs*_*protein*_) was more fully investigated in the following experiment by varying concentration of HA, both in PBS and soil extracts.

**Table 3:** Regression coefficients of Abs_protein_ as f(BSA) in the three contrasting matrices, also in the presence of citrate.

Analytical matrix	Slope of Abs protein as f [BSA]	s.e.
PBS	0.005573	0.000076
Soil extract in PBS	0.004485	0.000076
PBS spiked with HA (160 ppm)	0.004399	0.000076

### 3.2 Comparison of accuracy between Bradford and Lowry estimations of standards in PBS

**Fig. 2:**
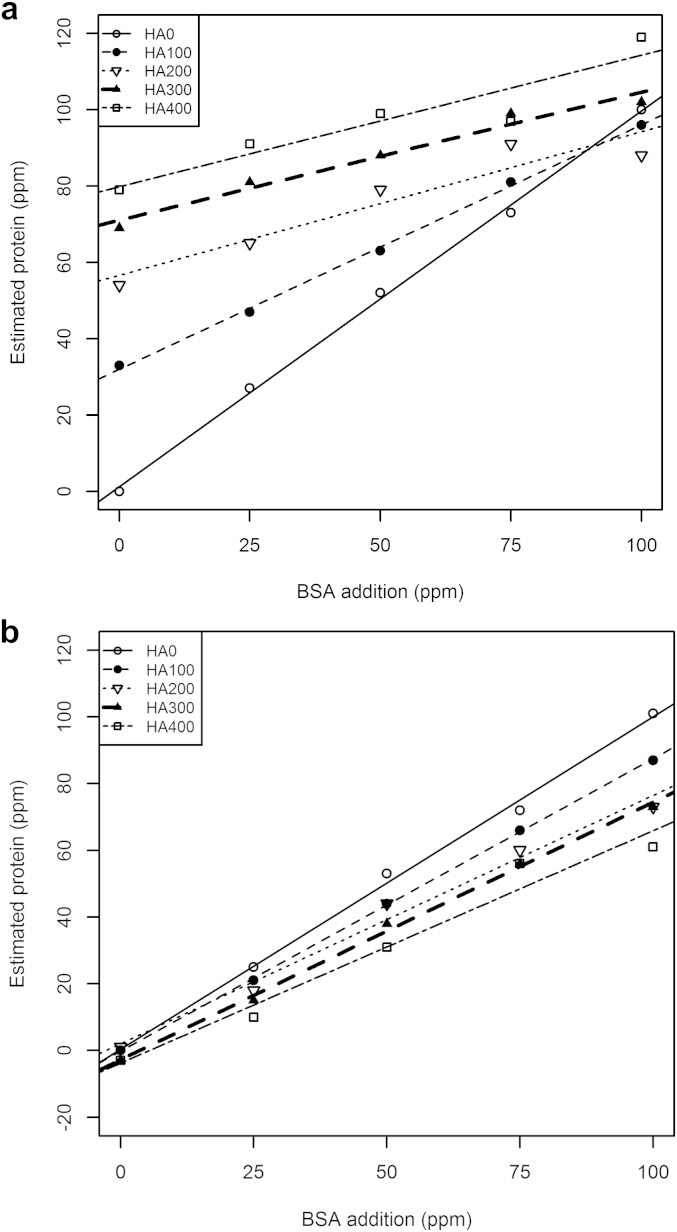
a) Bradford and b) Lowry estimation of protein as a function of humic acid and protein additions to buffer alone (HA0, 100, 200, 300 and 400 correspond to humic acid additions of 0, 100, 200, 300 and 400 ppm, respectively).

The modified two-to-three-reagent Lowry system was less time-sensitive than the Bradford thus permitting full use of the 96 well plates. The Lowry-based analytical procedure (including mixing of stock reagents) was achieved in approximately one hour. With the Bradford assay, addition of HA in the complete absence of protein resulted in a false positive estimation of protein (80 ppm protein estimated in the presence of 400 ppm HA) (Fig. 2a, *x* = 0). Although increasing inclusions of HA caused an additive effect in terms of total absorbance, an increasingly large underestimate of the additional protein also occurred, as seen by the reduction of slope angle with increasing HA content. For example, with 400 ppm of humic acid (Fig. 2a, HA400), the slope of linear regression is 65% less than in the absence of humic acid (HA0).

The Lowry microplate method gave more accurate estimates of the quantity of protein added (Fig. 2b). With the Lowry microplate assay, addition of HA alone did not result in a false positive indication of protein (Fig. 2b, *x* = 0). When combined with protein additions, a systematic underestimate of protein occurred (***P < 0.001). However, this was less than half the suppressive effect seen using the Bradford assay (only a 31% decrease in slope comparing regressions for HA400 and HA0; Fig. 2b).

Using the Bradford method, the mean squared errors of prediction (MSEP) of protein added to buffer (affected by additions of HA) were calculated using the method of Wallach and Goffinet (1987). MSEP increased with HA addition, from 2, to 364, 1152, 1983, and 2778, for HA additions of 0, 100, 200, 300 and 400 ppm, respectively. Using the Lowry-microplate technique, the observed values (protein measured) differ much less from the expected (protein added), with corresponding MSEP's of 2, 81, 219, 290 and 469, respectively. Thus with increasing additions of HA, the MSEP of the Lowry microplate technique was more than 5-fold smaller than the Bradford assay (Table S1).

### 3.3 Comparison between Bradford and Lowry estimations of standard additions to soil extract

**Fig. 3:**
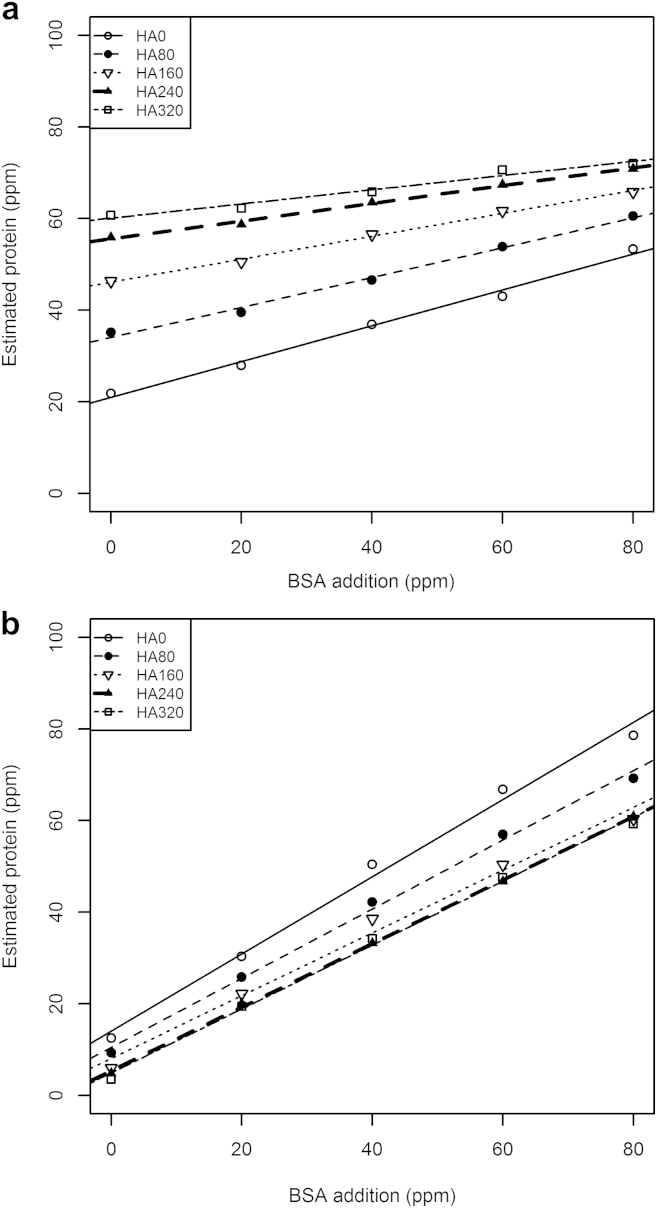
a) Bradford and b) Lowry estimation of protein in soil extract (soil 1) with buffer including analytical responses to additions of BSA and HA.

The protein concentrations in diluted extracts of soil 1 given by the Bradford and Lowry microplate methods (Fig. 3a and b, respectively), without BSA addition (*x* = 0) were 21.8 ppm and 12.5 ppm (50 and 100 μL assay concentrations, respectively). These absolute measurements, inclusive of soil protein, with a range of BSA and HA additions show response patterns similar to those observed with PBS alone (Fig. 2a and b). Using the Bradford assay (Fig. 3a), the small addition of humic acid (80 ppm) caused an increase in protein estimate of about 75%. In contrast, with the modified Lowry assay (Fig. 3b) the same quantity of humic acid caused a *decrease* in soil–extract protein estimations of about 25%.

### 3.4 Comparison of estimation accuracy between Bradford and Lowry

**Fig. 4:**
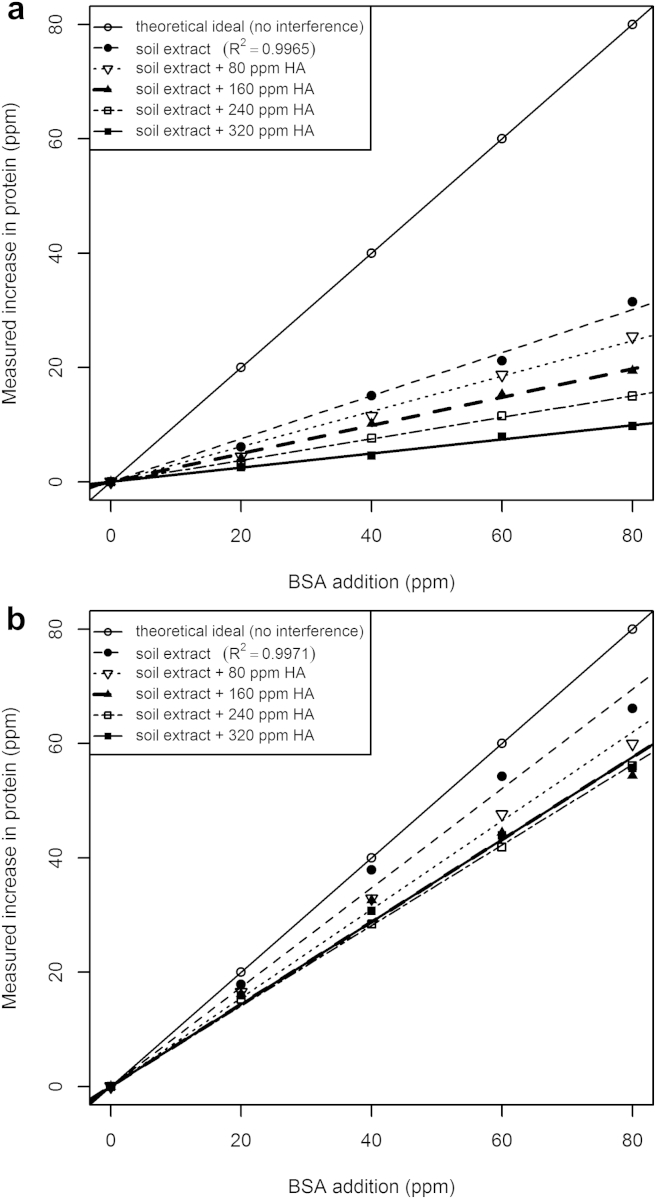
Polyphenol (HA) suppresses colour development from protein in a) Bradford and b) Lowry analyses of soil 1 extract.

Using extracts of Soil 1, the *increases* in protein estimate in response to known additions of BSA are presented in Fig. 4(a and b). Colour development due to pre-existing soil protein or directly from the added polyphenol is excluded. By comparison to the ‘theoretical ideal’ (1:1 line), using estimates given by the Bradford assay (Fig. 4a), substantial suppression of colour development due to protein occurs, even for soil extract with no HA addition. This would be especially problematic if quantifying extract protein using a calibration curve generated in PBS (common procedure). With the Bradford assay, linear regressions of the responses to added protein, by comparison to the 1:1 theoretical ideal, show underestimations of protein additions equal to 62, 69, 75, 81, and 86% (s.e. ± 0.8%) for 0, 100, 200, 300 and 400 ppm HA additions respectively.

In contrast, using the Lowry microplate assay (Fig. 4b), the underestimation of additions was substantially less, with corresponding underestimations of 13, 23, 28, 30, and 28% (±1.8%) for 0, 100, 200, 300 and 400 ppm HA additions respectively. Protein estimates assayed in response to BSA additions responded in a similar way between HA spiked PBS buffer and all 3 extracts of soil.

### 3.5 Assumptions of linearity

**Fig. S2:**
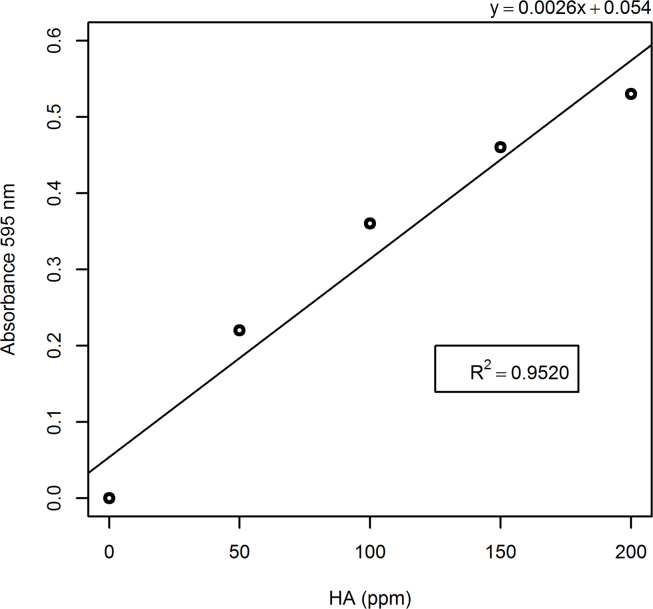
a) Linear, and b) 3rd order polynomial regressions of absorbance responses to humic acid additions in the Bradford assay.

Whereas linear regressions of absorbance increases using the Bradford assay accounted for 99.65% of the variance in response to protein additions (Fig. 4a), with HA additions, only 95.20% of the variance can be accounted for by the straight line model (Fig. S2a), with 99.99% being described by a 3rd order polynomial (Fig. S2b).

**Fig. S3:**
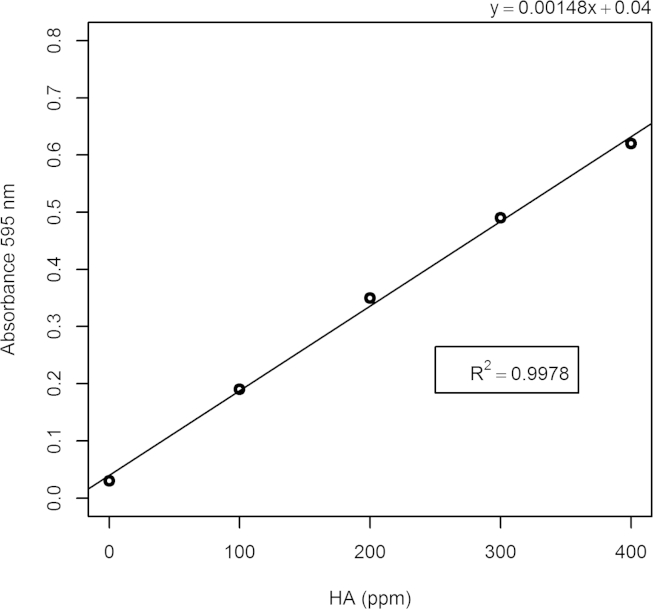
a) Linear, and b) 2nd order polynomial regressions of absorbance responses to humic acid additions in the Lowry assay.

Similarly, linear regressions of absorbance increases with the Lowry microplate assay accounted for 99.71% of the variance in response to protein additions (Fig. 4b). However, although Lowry responses to HA additions to buffer are, strictly speaking, best described by a 2nd order polynomial (Fig. S3b), 99.78% of the variance due to HA additions can be accounted for by a simple straight line (Fig. S3a). The Lowry microplate assay thus gave a more linear response than the Bradford assay to both polyphenol and protein additions to soil extracts.

### 3.6 Estimates of soil protein content

Estimates calculated for all extracts (corrected for dilution) are presented in Table 4 for reference. It is important to remember that although colour development from protein *per se* using the Bradford assay is more suppressed by the presence of polyphenol, it is variably compensated for by the colour development directly from the polyphenol itself. This most likely explains the apparent overestimation of protein in soil 3. In contrast, there appears to be negative bias in soils 1 and 2 by comparison to concentrations given by the Lowry assay. This is most likely due to suppression of colour development from protein as demonstrated in Fig. 4a, i.e. where the protein fraction is proportionally larger, the Bradford assay underestimates. Extracts of Soil 1 and Soil 2 (arable, and fallow managements, respectively) exhibit lower polyphenol/protein ratios than extract of Soil 3 (grassland).

**Table 4:** Extract protein concentrations (corrected for dilution). Modified Lowry also provides estimate of phenolics (as HA equivalent: ‘HAE’).

Extract	Soil 1		Soil 2		Soil 3	
	Protein estimate (ppm)	Polyphenol estimate (ppm)	Protein estimate (ppm)	Polyphenol estimate (ppm)	Protein estimate (ppm)	Polyphenol estimate (ppm)
Bradford	163.7 ± 2.4^a^	–	33.1 ± 0.3	–	374.9 ± 2.5	–
Lowry	187.5 ± 3.3	1048 ± 11	49.0 ± 0.5	274 ± 2	328.8 ± 10.6	3384 ± 11
HAE/Prot ratio	5.6		5.6		10.3	

a ± Indicates standard error.

## 4 Discussion

With regard to assayed protein content (Table 4), although colour development from protein *per se* using the Bradford assay was found to be highly suppressed by the presence of polyphenol (Fig. 4a), this was variably (and nonlinearly) compensated for by the colour development directly from polyphenol complexes with the Bradford dye (Fig. S2b). This most likely explains the apparent overestimation of protein in soil 3 by the Bradford assay in comparison to the Lowry. Overestimation was seen previously with soil extracts containing a large phenolic fraction e.g. Whiffen et al. (2007). The increased HAE/protein ratio indicated by the Lowry assay for soil 3 is in agreement with the findings of Martens et al. (2004) where the proportion of organic C present as phenolics in grassland was also greater than that in arable soil: grasslands are highly competitive, with phenolics being produced biologically as competitive phytotoxins of allelopathy (Lipinska and Wanda, 2005) and as signalling agents between roots and rhizobia (Cesco et al., 2012), most likely explaining the high HAE/protein ratio found in the present study. The phenolic content of soil is of further contemporary interest as it has been linked to soil organic matter dynamics in the context of land-use change, climate, and CO_2_ emissions, e.g. (Fernandez et al., 2012).

Citrate extracts are best thought to contain a mixture of biochemicals from soil microbes, humified soil organic matter, and reaction products of extraction (Nannipieri and Eldor, 2009). In the current study, depending on soil type, estimates of polyphenol content (shown as HAE) were between 5 and 10 times greater than the protein content. Solid-state ^13^C DPMAS NMR spectra of various ‘GRSP’ extracts were presented by Schindler et al. (2007) showing high degree of aromaticity but little aliphatic C (41%–51% and 4%–11%, respectively). The authors summarised this was indicative of a greater proportion of humified organics as opposed to protein content with BSA showing only 12% aromaticity, and 54% aliphatic content. The protein/HAE ratios we observed thus sit comfortably in the range suggested by NMR.

It is not possible to comment precisely upon the accuracy of the determinations of soil extract protein *per se* (soil extract in the absence of any BSA or polyphenol additions), because there is currently no universally accepted method to measure protein in soils, with each method being subject to idiosyncratic artefacts (Nannipieri and Eldor, 2009). Therefore, in this study, comparison of accuracy is based upon the assumption that BSA, the most commonly used protein reference standard, is a good model for soil protein. Although questions have been raised with regard to the suitability of using a non-microbial protein as a proxy for protein in soils (Criquet et al., 2002), BSA remains the most frequently used model, e.g. Taylor and Williams (2010) and Young et al., (2012) and to our knowledge no replacement is receiving much consideration. The well characterised BSA standard (Wu et al., 2011) is thus still used extensively as a reference protein.

It is now widely recognised that the extraction procedure of Wright and Upadhyaya (1996) extracts large quantities of non-mycorrhizal proteins from soil with even heat-labile proteins contributing to measures of GRSP (Janos et al., 2008, Rosier et al., 2006). However, Bradford determinations of ‘GRSP’ or ‘Bradford reactive soil protein’ (BRSP) are still commonplace, and good correlations are repeatedly found with aggregate stability and soil organic C and N, e.g. Emran et al. (2012). It follows that *if* the Bradford reactive fraction is to serve as a surrogate measure of aggregate stability, or organic matter content, then the *de facto* GRSP measure may still be useful. However, if the motivation is a better understanding of soil organic matter dynamics, or if we are to continue to estimate protein from extracts (and not only citrate extracts) then simple analytical techniques that are more selective for ‘proteinaceous’ vs. more ‘polyphenolic’ material will be more descriptive. Moreover, confounding these pools through use of the Bradford assay will cloud interpretations of the respective contributions of these organic fractions to soil properties.

It is likely that in future, a rapid measure of soil protein distinct from highly humified pools will be called for, e.g. if we are to attempt to quantify the impacts of microbial protein found within extracellular matrices of entire microbial communities, and not misleadingly attributing extractable protein and humified organic matter collectively to AMF. Extracellular microbial proteins are produced *in vivo* with a variety of suspected impacts upon soil physical properties (Or et al., 2007). The structural roles of extracellular proteins are currently being explored in related scientific disciplines and are thought to help impart strength and elasticity to biofilms (Flemming and Wingender, 2010). These extracellular polymers, produced by the living soil biomass (including AMF, saprophytic fungi, bacteria and archaea) are thought to improve aggregate stability, weight for weight, to a greater extent than total SOM (Chenu, 1993, Roberson and Firestone, 1992, Tang et al., 2011, Watts et al., 2005). The same was also originally hypothesised for glomalin (Wright and Upadhyaya, 1998) and links between confounded soil protein/polyphenolic pools and aggregate stability were since reported by many studies. A firm causal link between AMF and aggregate stability was later established through other methods, e.g. Rillig et al., (2010). A greater understanding of community-wide extracellular proteinaceous material in soils is now required, and besides improved specificity of extraction methods, the single most important step is likely to be avoiding the largest known artefact currently affecting colorimetric analyses, i.e. the interference from polyphenolic content.

## 5 Conclusion

The modified Lowry assay presented here provided a reasonable estimate of polyphenolic content and a more accurate estimate of protein content in citrate extracts of 3 contrasting soils, and model extracts. It is therefore of potential value in comparative studies of total extractable protein where the polyphenol content is expected to be high.

These findings lead us to add caution against loose usage of the terms ‘glomalin related soil protein’ (GRSP) and ‘Bradford reactive soil protein’ (BRSP) to describe the ‘Bradford reactive fraction’ (BRF) in soil extracts, which itself produces a highly complex analytical response affected by the HAE/protein ratio.

## Figures and Tables

**Figure:**
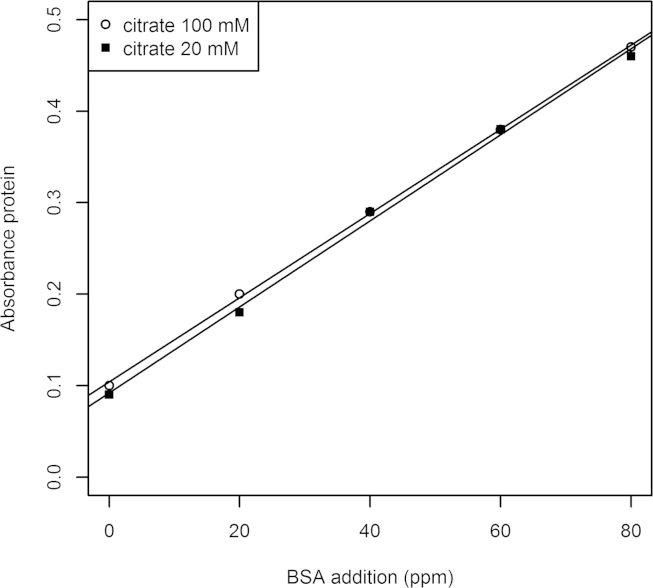


**Figure:**
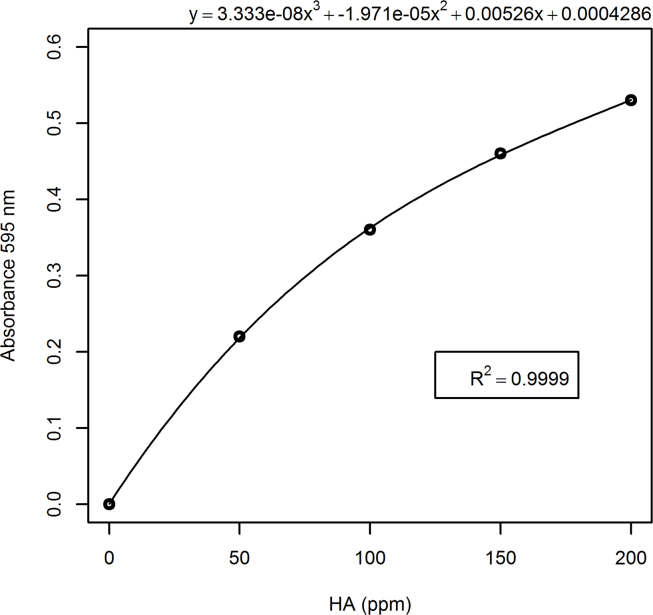


**Figure:**