# Supplementary material for: A comparison of two colorimetric assays, based upon Lowry and Bradford techniques, to estimate total protein in soil extracts
Source: Soil Biol Biochem. 2013 Dec;67:166–73. doi: 10.1016/j.soilbio.2013.08.017 (PMC3819989; doi:10.1016/j.soilbio.2013.08.017)
Supplement: Supplementary file 1 [file mmc1.docx]

Table S1: Observed minus expected measurements of protein in buffer with HA additions.

| Expected protein  (BSA added [ppm]) | HA conc (ppm) | Observed Bradford  (ppm) | Observed Lowry  (ppm) | Bradford  O-E^a^ | Bradford  (O-E)^2^ | Lowry  O-E | Lowry  (O-E)^2^ | Bradford  MSEP^b^ | Lowry MSEP |
| --- | --- | --- | --- | --- | --- | --- | --- | --- | --- |
| 0 | 0 | 0.0 | 0.0 | 0.0 | 0 | 0 | 0 | 2 | 2 |
| 25 |  | 27.2 | 25.1 | 2.2 | 5 | 0 | 0 |  |  |
| 50 |  | 51.5 | 52.5 | 1.5 | 2 | 3 | 6 |  |  |
| 75 |  | 73.3 | 74.0 | -1.7 | 3 | -1 | 1 |  |  |
| 100 |  | 100.0 | 99.5 | 0.0 | 0 | -1 | 0 |  |  |
| 0 | 100 | 33.3 | 0.2 | 33.3 | 1110 | 0 | 0 | 364 | 81 |
| 25 |  | 46.9 | 19.6 | 21.9 | 481 | -5 | 29 |  |  |
| 50 |  | 63.4 | 45.4 | 13.4 | 180 | -5 | 21 |  |  |
| 75 |  | 80.6 | 65.2 | 5.6 | 31 | -10 | 97 |  |  |
| 100 |  | 95.9 | 84.0 | -4.1 | 17 | -16 | 256 |  |  |
| 0 | 200 | 54.3 | 0.3 | 54.3 | 2951 | 0 | 0 | 1152 | 219 |
| 25 |  | 65.0 | 17.9 | 40.0 | 1603 | -7 | 50 |  |  |
| 50 |  | 78.6 | 41.3 | 28.6 | 817 | -9 | 75 |  |  |
| 75 |  | 90.7 | 58.9 | 15.7 | 248 | -16 | 258 |  |  |
| 100 |  | 88.1 | 73.3 | -11.9 | 142 | -27 | 713 |  |  |
| 0 | 300 | 69.3 | -2.6 | 69.3 | 4802 | -3 | 7 | 1983 | 290 |
| 25 |  | 80.8 | 14.8 | 55.8 | 3115 | -10 | 105 |  |  |
| 50 |  | 87.8 | 36.8 | 37.8 | 1428 | -13 | 175 |  |  |
| 75 |  | 98.8 | 52.4 | 23.8 | 565 | -23 | 512 |  |  |
| 100 |  | 102.4 | 74.5 | 2.4 | 6 | -26 | 651 |  |  |
| 0 | 400 | 79.1 | -1.7 | 79.1 | 6260 | -2 | 3 | 2778 | 469 |
| 25 |  | 91.2 | 12.2 | 66.2 | 4384 | -13 | 164 |  |  |
| 50 |  | 98.8 | 31.6 | 48.8 | 2386 | -18 | 340 |  |  |
| 75 |  | 97.4 | 55.7 | 22.4 | 503 | -19 | 371 |  |  |
| 100 |  | 118.8 | 61.7 | 18.8 | 355 | -38 | 1469 |  |  |

^a^O-E = Observed minus Expected

^b^MSEP = mean squared error of prediction
